# Tracking of Short Distance Transport Pathways in Biological Tissues by Ultra-Small Nanoparticles

**DOI:** 10.3389/fchem.2018.00028

**Published:** 2018-03-23

**Authors:** Jana S. Segmehl, Alessandro Lauria, Tobias Keplinger, John K. Berg, Ingo Burgert

**Affiliations:** ^1^Wood Materials Science, Institute for Building Materials, Department of Civil, Environmental and Geomatic Engineering, ETH Zürich, Zurich, Switzerland; ^2^Bio-inspired Wood Materials, Applied Wood Materials, EMPA, Dübendorf, Switzerland; ^3^Laboratory for Multifunctional Materials, Department of Materials, ETH Zürich, Zurich, Switzerland

**Keywords:** wood tissue, transport pathways, Raman microscopic imaging, X-ray diffraction, hafnia, nanophosphors

## Abstract

In this work, ultra-small europium-doped HfO_2_ nanoparticles were infiltrated into native wood and used as trackers for studying penetrability and diffusion pathways in the hierarchical wood structure. The high electron density, laser induced luminescence, and crystallinity of these particles allowed for a complementary detection of the particles in the cellular tissue. Confocal Raman microscopy and high-resolution synchrotron scanning wide-angle X-ray scattering (WAXS) measurements were used to detect the infiltrated particles in the native wood cell walls. This approach allows for simultaneously obtaining chemical information of the probed biological tissue and the spatial distribution of the integrated particles. The in-depth information about particle distribution in the complex wood structure can be used for revealing transport pathways in plant tissues, but also for gaining better understanding of modification treatments of plant scaffolds aiming at novel functionalized materials.

## Introduction

Short distance transport is crucial in the metabolism of living plants as well as for the functionalization of biological tissues for the development of bio-inspired and bio-based materials. In terms of specific and non-specific targeting of enzymatic structures in cells, novel designed bio-labels applied in electron microscopy and tomographic techniques can be used to improve the understanding of metabolic pathways and organizational structures of cell organelles (Geimer, 2009; Mayhew, 2011; Wang S. G. et al., 2012; Wang Z. Y. et al., 2012; Deng et al., 2014). However, most established marker systems applied in soft biological tissues, e.g., fluorescent proteins or nanoparticles for immuno-gold-labeling, do target specific molecules in structural assemblies or are not sufficiently small enough to diffuse into the nano-pores of many rigid biological materials (Daniel, 1994; Hill and Papadopoulos, 2001; Miyawaki et al., 2003; Daniel et al., 2004; Retterer and Simpson, 2012). Hence, their application in the analysis of nano-porous biological materials is limited to the post-preparative staining of thin sections (Ruel et al., 2006), whereas for *in situ* staining of nano-sized features inside the native structure and accessing the respective pathways, a diffusion-driven infiltration of the hydrated structure with detectable and small markers is needed.

Light microscopy and further characterization techniques, including electron microscopy, super-resolution microscopy techniques, and various scattering methods, allow for resolving the structural assembly of natural materials from the macroscopic level down toward the cellular and subcellular scale (Hell, 2007; Fruh et al., 2015). However, the complex organic nature and the hydrated native state of biological materials still present challenges for operating electron microscopy and tomography at high-resolution because of sample degradation under a high energy electron beam. The deterioration of organic matrices can be limited by standard preparation techniques, like dehydration, fixation and electron rich staining, though substantial alterations of the natural tissue organization cannot be excluded (Walther and Muller, 1999; Jansen et al., 2008; Beecher et al., 2009).

Recently, a wide range of inorganic ultra-small particle systems with narrow size distribution and tailorable functionalities have become available due to remarkable progress in the synthesis of functional nanomaterials (Cushing et al., 2004; Niederberger and Pinna, 2009). Their designable optical properties, crystallinity, and high electron density make them optimal candidates for universal tracker systems for the analysis of porous biological materials (Howes et al., 2014).

**Figure 1:**
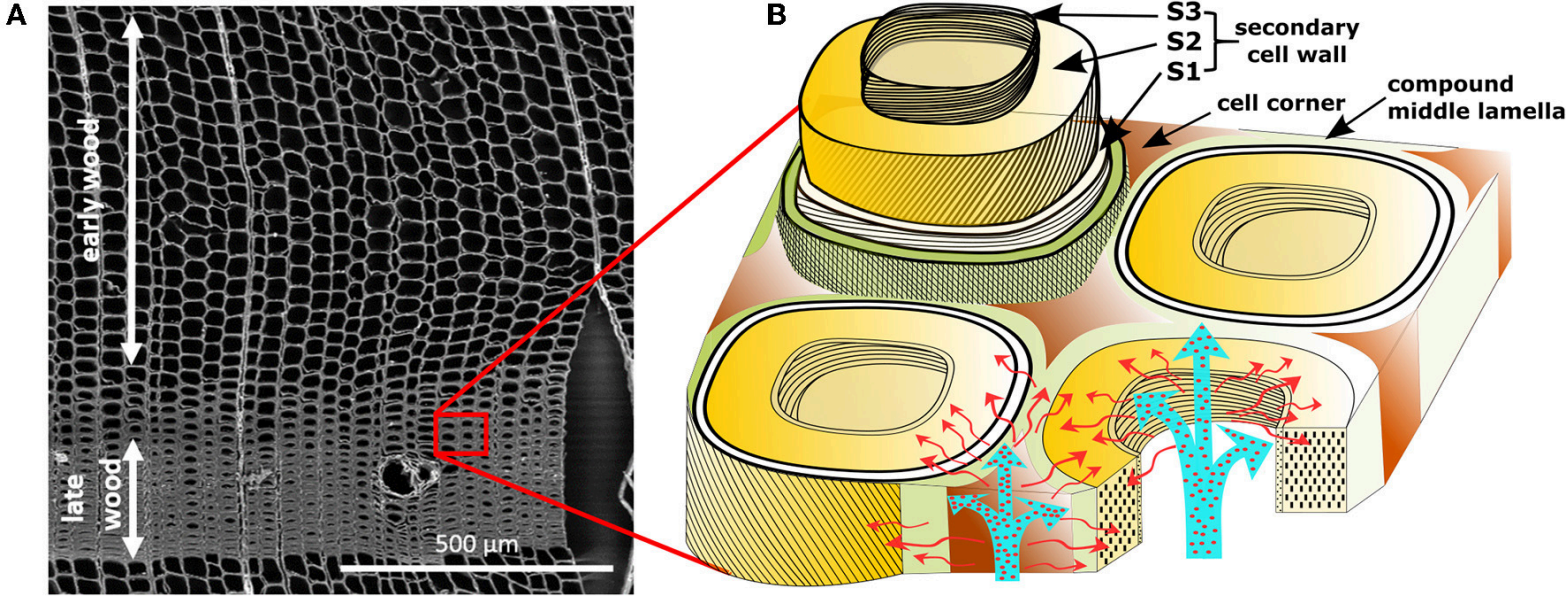
Micro- and ultrastructure of wood. **(A)** SEM overview of honeycomb-like structure of spruce wood cells (tracheids) in the cross section. Early wood shows larger lumina, while late wood present higher cell wall/lumen volume ratios. **(B)** Cell wall model with potential diffusion pathways of aqueous media through the lumina and nano-porosity present in the cell wall. Cells are connected by a compound middle lamella (middle lamella + primary cell wall). The secondary cell wall consists of distinct layers, S1, S2, and S3, differentiated through chemical composition and cellulose microfibril angle.

In this work, short distance pathways and particle distributions were studied in wood as a biological model system, characterized by a complex hierarchical structure based on the arrangement of its macromolecular organic constituents, cellulose, hemicelluloses, and lignin (Rowell, 1984). Layers of parallel aligned cellulose fibrils embedded in a matrix of lignin and hemicelluloses form the secondary cell wall, a main contributor to the structural support of the tubular wood cells Figure 1A) (Salmen and Burgert, 2009). A highly lignin enriched lamella surrounds the cells on the outside (middle lamella and cell corner) and connects them to an anisotropic cell assembly (Figure 1B). The specific porosity of these cell wall layers and its contribution to transport pathways in the living tree and for post-processing (wood modification) are still not fully understood (Figure 1B) (Hill et al., 2004). Gas phase porosity measurements and solute exclusion are suitable techniques for the determination of the accessible inner surface and pore size distribution, but do not provide any information on material penetration and the spatial distribution of the pores. Recently, Raman hyperspectral mapping was proposed for label-free analysis of the cellular composition and a spatial reconstruction based on chemical information (von Erlach et al., 2015; Hedegaard et al., 2016).

Here, we used europium-doped hafnium dioxide particles with ultra-small size and narrow size distribution (3.0 ± 0.4 nm average linear dimension), for the investigation of the percolation pathways and nano-porosity in the native wood structure (Lauria et al., 2013). This was previously determined by solute exclusion measurements to be less than 5 nm (Hill et al., 2005), which can be seen as a rough threshold for the size of a nanoparticle to be able to enter the wood cell wall. In this context, the work of Lauria et al. (2013) revealed that the solvolytic synthesis of HfO_2_ provides singly dispersed nanocrystals with slightly elongated shape, the longer axis of which is smaller than 4 nm. While their bright red luminescence under UV excitation was activated by incorporating rare earth ions as dopants, a distinct resonant fluorescence of moderate intensity, obtained by illuminating with green laser light, make the particles detectable in confocal Raman microscopy (D'Aléo et al., 2007). In contrast to metallic particles used in surface enhanced Raman spectroscopy (SERS), the laser induced photoluminescence of the applied particles allows for the simultaneous and constant recording of biochemical information and distribution patterns of the nanoparticles. Moreover, because of the high stability of these particles in aqueous media, these dispersions enable the study of biological materials under native conditions. Indeed, the hierarchical structure of wood with a broad size distribution of voids, ranging from the nano- up to the micron-scale, is an ideal material system to study the feasibility of tracker systems in natural porous materials, based on diffusion driven infiltration with nanoparticles, which is usually limited by the native percolated porosity present in the hydrated material. Although transport pathways in plants and the uptake of nanoparticles into different tissues was studied in various biological systems on the macro scale, little is known so far about the interactions at the nanoscale (Marmiroli and White, 2016). With this system a detailed knowledge on nutrition transport and water conductance in the organism can be gained.

## Materials and methods

### Materials

Norway spruce (*Picea abies*) wood was cut and prepared to 5 × 5 × 5 mm^3^ cubes with smooth surfaces. Hafnium (IV) tert-butoxide (99.99% + Zr) was purchased from Multivalent Laboratory, Eriswell, UK. Europium (III) acetate hydrate (99.99%), 2-[2-(2-methoxyethoxy)ethoxy]acetic acid (MEEAA, technical grade), and benzyl alcohol anhydrous (BnOH, 99.8%) were purchased from Sigma-Aldrich. All precursors were used as received.

### Synthesis of Eu:HfO_2_ nanoparticles

The synthesis of monoclinic HfO_2_ nanoparticles by non-aqueous sol-gel chemistry was carried out according to a previous work in a glovebox (O_2_ and H_2_O < 0.1 ppm) (Lauria et al., 2013). Briefly hafnium (IV) t-butoxide was added to anhydrous benzyl alcohol (BnOH) into a glass test tube together with the appropriate volume of europium acetate pre-dissolved in BnOH. The reaction mixture was transferred into a Teflon liner of 45 mL, slid into a steel autoclave and carefully sealed. The autoclave was taken out of the glovebox and heated in a furnace at 220°C for 4 days. The resulting suspension was centrifuged and thoroughly washed with diethyl ether (Aldrich). The washed particles were re-dispersed in ethanol and functionalized by the addition of MEEAA (2-[2-(2-methoxyethoxy)ethoxy]acetic acid. After the solution turned clear under magnetic stirring, hydro-alcoholic (around 10 %/v/v ethanol) stable suspension with a concentration of 5 mg/mL was obtained by dilution with water. The full characterization of this particle system was conducted previously by Lauria et al. (2013).

### Nanoparticle infiltration

The cubes of Norway spruce wood were placed into a flask and stored under water for 2 days to reach water saturation over the whole sample volume and facilitate the particle infiltration process. Afterwards, the water was exchanged by a hydro-alcoholic stable solution containing the pre-synthesized nanoparticles at a concentration of 5 mg/mL. After 6 days of incubation, the samples were washed with deionized water for 24 h. Finally, the samples were dried under controlled conditions in a climate room (20 °C/65 % r.h.) until a constant mass was reached.

### Scanning electron microscopy

Smooth faces of the wood cubes approximately 2 mm below the surface in both-, cross sectional and longitudinal direction (LT-plane) were prepared using a rotary microtome. The measurements were performed on the block samples using a FEI Quanta 200 probe in the low vacuum mode, driven at an accelerating voltage of 20 kV equipped with a backscattered electron and secondary electron detector.

### Raman analysis

Twenty micrometer-thick cross sections from untreated and particle solution incubated cubes were cut with a rotary microtome (RM 2255; Leica Germany), in a position approximately 1 mm below the sample surface. The cross-sections were sealed on a glass slide in wet conditions (deionized water) with a cover slip (thickness: 0.17 mm). For further details on the sample preparation see (Gierlinger et al., 2012).

Raman spectra were recorded in backscattering configuration through an inVia Raman microscope (Renishaw, UK) equipped with a motorized xyz stage using the 532 nm line of a Nd:YAG laser. A 100 x oil immersion objective with numerical aperture (NA) 1.3 (Nikon) was used. The laser was focused with a diffraction limited spot size of 0.61 × λ/NA onto the samples and the Raman light was detected by an air-cooled charge coupled device (CCD) camera behind a spectrometer (inVia) with a spectral resolution of about 1 cm^−1^. The mapping was achieved with a step size of 300 nm and an integration time of 0.15 s in the spectral region between 1050 and 2650 cm^−1^. Wire 4.1 software (Renishaw) was used for measurement setup and spectral pretreatment. Data analysis was performed using CytoSpec (version 2.00.01), a commercially available MATLAB based software. For intensity profile line scans, the spectra were baseline corrected and the particles fluorescence relative intensities were determined as signal intensity at 2480 cm^−1^. Hierarchical Cluster Analysis (HCA) was performed on Raman maps without baseline correction. The spectral region was chosen to be 1800–2700 cm^−1^ for the particle signal and 1100–1700 cm^−1^ for the finger print region of the wood, respectively. For the cluster analysis, the distance matrix was calculated by the *D*-value method and the Ward's algorithm was used to perform the clustering.

### Synchrotron scanning WAXS measurement

Scanning wide-angle X-ray scattering (WAXS) measurements were carried out at the nano-focus end-station P03 (MINAXS) of the synchrotron beamline PETRA III (DESY, Hamburg) (Krywka et al., 2012). The X-ray beam (17 keV, 0.7293 Å) was focused to a spot size of 1 μm by 1 μm using two-dimensionally confining hard X-ray silicon waveguides, as described elsewhere (Krywka et al., 2012, 2013). Due to the low signal intensity, 2D diffraction patterns were recorded with a single-photon counting PILATUS 1 M (981 × 1043 pixels, 172 × 172 μm^2^) detector. Samples were mounted on a sample holder fixed on a hexapod to ensure accurate alignment with an optical microscope installed at the experimental end-station. The sample-detector distance was set to 17.7 cm to give a maximum q-space ≈ 37.6 nm^−1^. A scanning step size of 1 × 1 μm^2^ and an exposure time of 1 s helped avoiding X-ray radiation damage of the cellulose. Data analysis was automated using macros in the software Fit2D (V16.041). A background reference frame was subtracted from all 2D patterns to account for air scattering. The sample-detector distance, beam center and detector tilt was calibrated against a LaB_6_ standard by the calibrant routine implemented in Fit2D.

For the reference spectra, all measurements were performed on an Empyrean diffractometer (equipped with a PIXCel1D detector) from PANalytical (Netherlands). The X-ray diffraction (XRD) measurements for HfO_2_ and spruce powder (measured over Si zero background sample holders) were performed in reflection mode (Cu Kα radiation at 45 kV and 40 mA), operated with 0.026° step size, and time per step of 75.2 s. Crystalline cellulose in the wood cellular structure is referenced to cellulose Iβ (monoclinic, P21 space group, unit cell: a = 7.784 Å, b = 8.201 Å, c = 10.380 Å, and γ = 96.5°) (Li et al., 2016).

## Results

**Figure 2:**
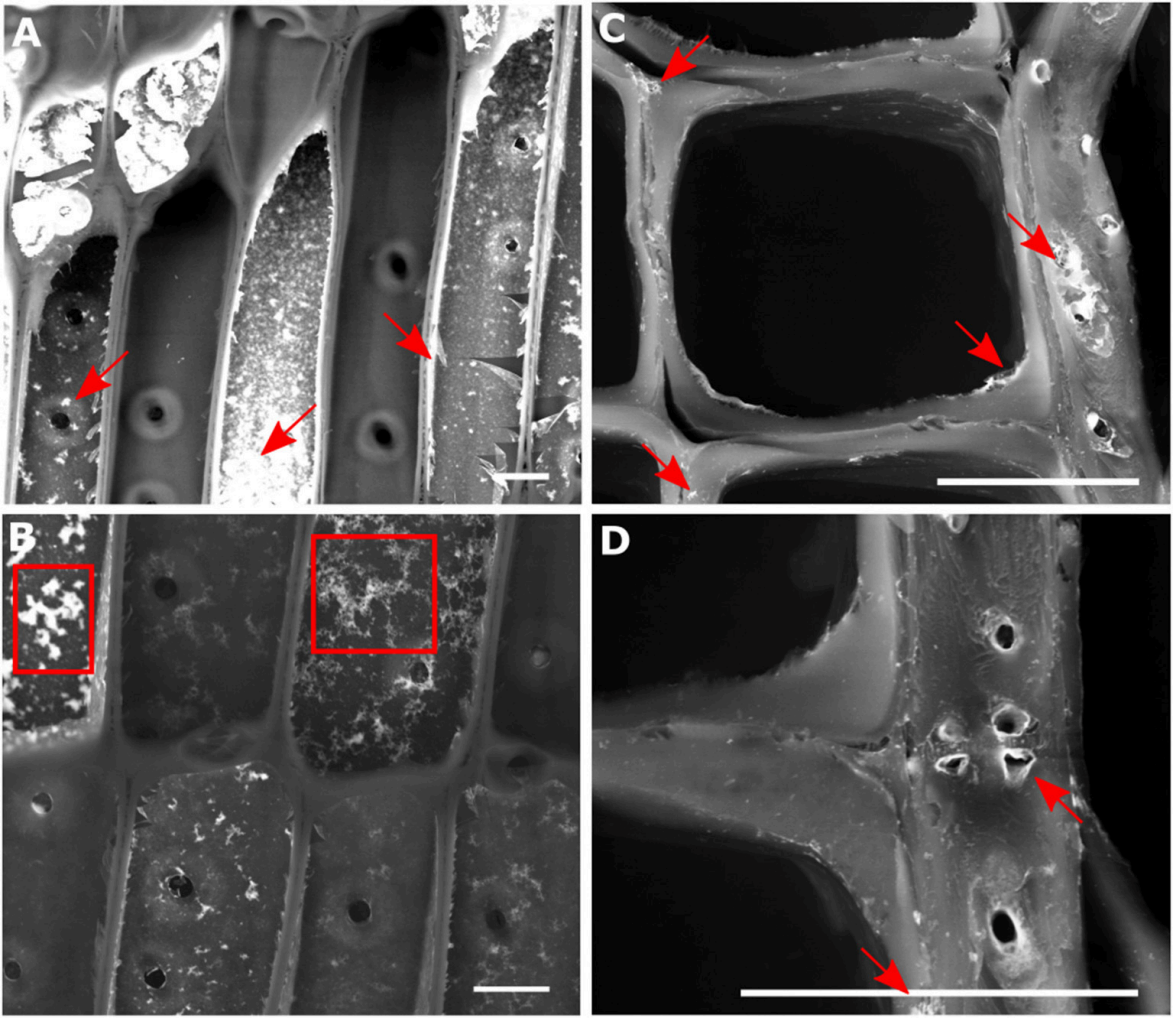
Backscattered electron micrograph of a longitudinal tangential (LT) cut **(A,B)** and cross section **(C,D)** through spruce wood infiltrated with Eu:HfO_2_ nanoparticles. Agglomerated (marked with arrows) and fractals [boxes in **(B)**] formed at the interface between lumen and cell wall are clearly visible, while isolated nanoparticles cannot be resolved. Scale bar = 20 μm.

Small cubes of spruce wood were infiltrated with a water stable suspension of europium-doped HfO_2_ nanoparticles. The particles were synthesized through a previously established solvolythic protocol, which was shown to lead to single dispersed crystals with round, slightly elongated, shape, with sharp size distribution and diameter of around 3–4 nm (Lauria et al., 2013). After being functionalized, these nanocrystals could be stabilized in water dispersions while retaining their fluorescence properties, provided by the doping with Eu^3+^ ions. Using water instead of organic solvents for the infiltration helps to retain the native ultrastructure of wood (Mantanis et al., 1994). The successful uptake of the particle sol after immersion for 6 days was studied using electron microscopy in back scattering configuration (Figure 2). This measurement, probing for Z-contrast, revealed, after drying, an inhomogeneous distribution of the nanoparticles throughout the whole sample volume. In several cells, no coating of hafnia particles at the lumina was observed (Figure 2A). Nevertheless, no gradient in hafnia concentration from the outside to the center of the wood cube was detected, suggesting a penetration of the particle sol through the whole sample thickness.

With an average diameter of 3 nm, individual Eu:HfO_2_ particles are too small to be visualized in the wood structure using SEM analysis (Walther and Muller, 1999). Therefore, only the presence of agglomerates at the interface and accumulations of particles in close vicinity to the cell wall tissue could be determined (Figures 2A,C,D, arrows). Single particles internalized in the nanopores cannot be resolved with this method. The particle coatings at the lumen side of the cell wall consists of clusters of single particles with a broad size distribution and different concentrations. The formed film appears to be rough and ruptured in various sites. In some areas, fractals formed on the surface can be observed (Figure 2B, boxes). With high probability, this film is deposited during the drying of the specimen after infiltration. During drying, solvent evaporation yields an increase in particle concentration and can destabilize the colloidal suspension of the hafnia particles. In proximity to the cell wall surface, the diffusion pathway of the particles is limited and, as they approach sufficiently close for interaction, aggregation into clusters can occur. Fractal-structured clusters can form on the surface following a diffusion limited growth process (Sander, 2000). As a result, cluster-cluster aggregation yields the formation of a collection of clusters with a broad size distribution at the interface between cell walls and cell lumina (Sattler, 2010).

**Figure 3:**
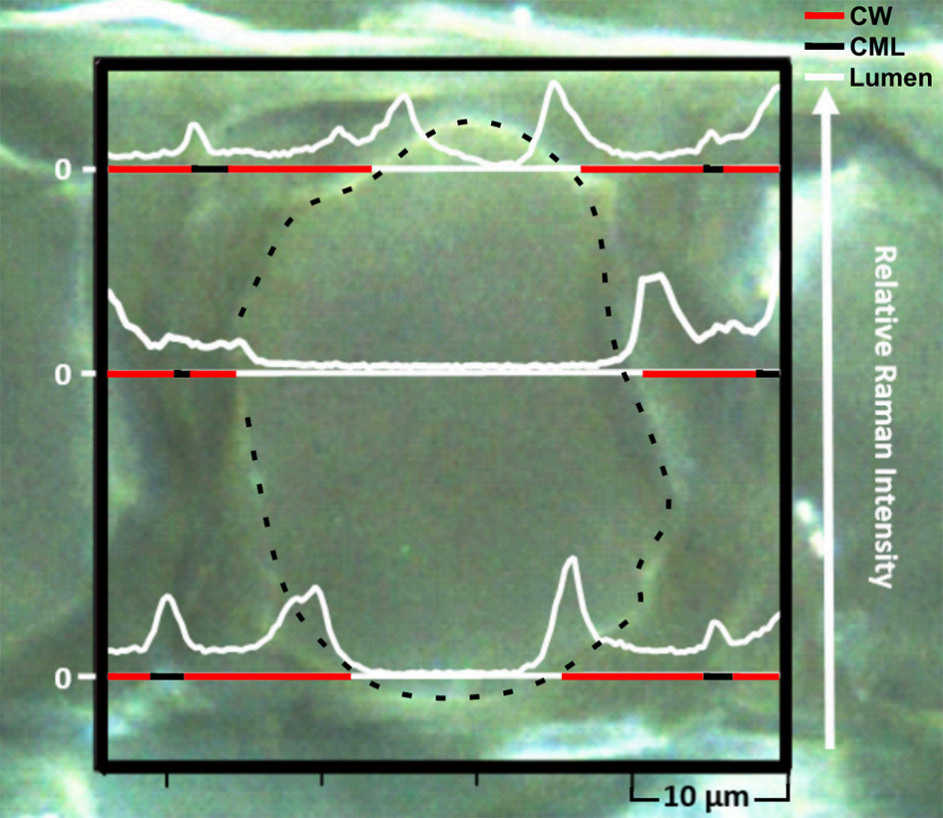
Microscope image overlaid with single lines extracted from the hyperspectral Raman mapping showing the resonance fluorescence intensity (2480 cm^−1^) profile of Eu:HfO_2_ nanoparticles infiltrated in the wood ultrastructure. (CW, cell wall; CML, compound middle lamella; dashed line, cell wall/lumen interface).

For a detailed characterization of the diffusion-controlled infiltration of nano-trackers into the wood cell wall, a Raman-based analysis was conducted by mapping thin sections taken at least 1 mm from the cube surface. Thus, one can make use of the characteristic luminescence, which is obtained by the europium doping of the nanoparticles. The peculiar photo-physical properties of the Eu:HfO_2_ nanocrystals are manifested in the rare earth based luminescence which is activated through the homogeneous incorporation of Eu^3+^ ions in the nanocrystal lattice. The resulting Eu^3+^ related emission can be excited by 532 nm laser irradiation, through the parity-forbidden ^7^F_0, 1_-^5^D_1_ transition (Lauria et al., 2013). Being of relatively higher intensity, compared to Raman scattering, the emission lines stimulated by laser irradiation represent a suitable tool for their independent detection, even at low concentration. An extraction of single lines from this map, plotting the intensity of the 2480 cm^−1^ band, which corresponds to the Eu^3+^ laser-stimulated red emission at 613 nm, reveals their local distribution in the wood cells and cell walls (Figure 3). The spectral position of these emission lines at relatively high wavenumbers reduces the overlap with the peaks coming from wood and therefore a simultaneous detection of both, particle fluorescence and chemical information of substrate, is possible.

Higher intensities of particle fluorescence were detected in the cell corners, the middle lamella region and along the interface between the inner lumen and the cell wall. In the S2 layer, the luminescence was present as well, but the detected signal was of lower intensity.

**Figure 4:**
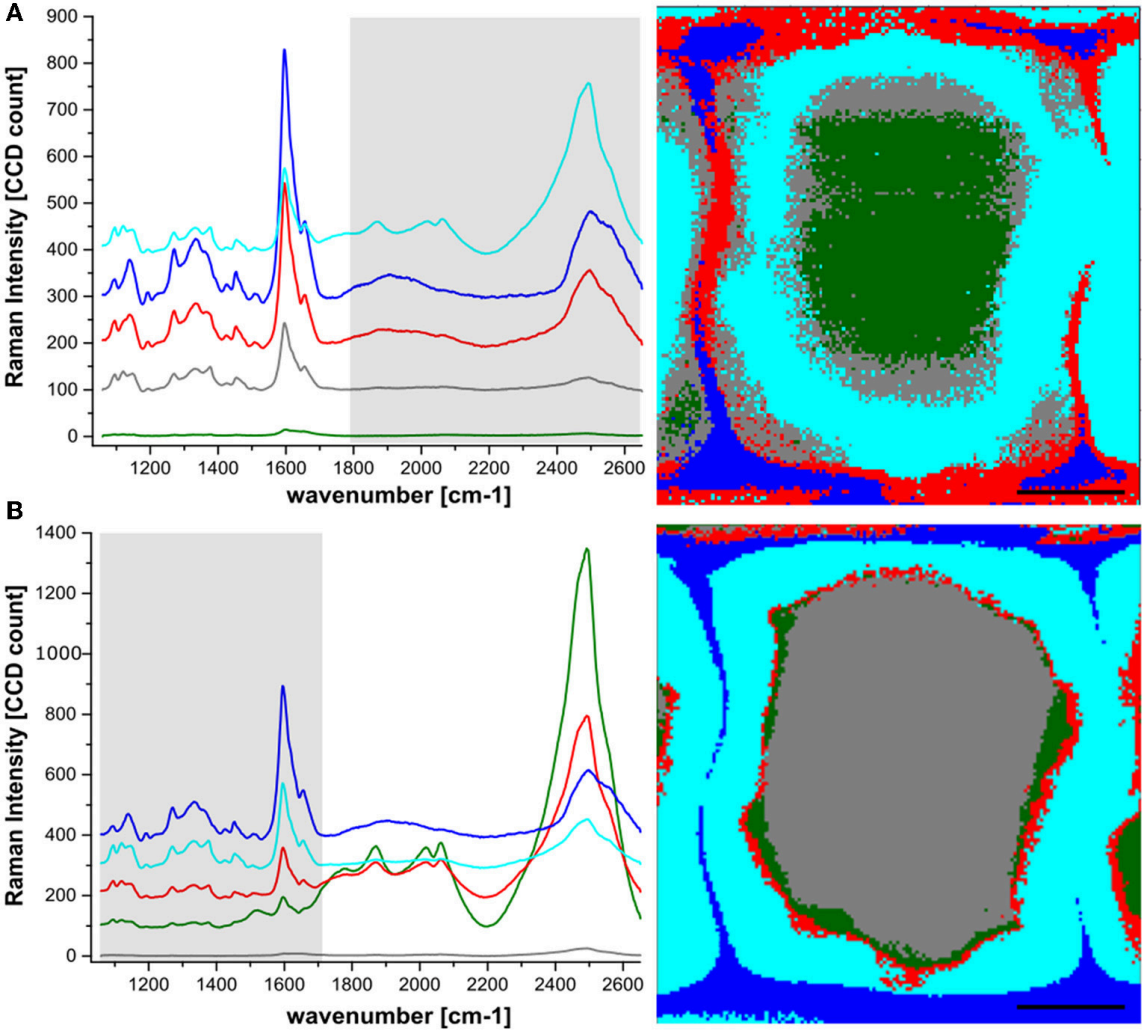
Hyperspectral Raman mapping of spruce wood incubated with Hafnia nanoparticles. HCA with 5 components was used for reconstruction, revealing the differences in the particle luminescence intensity **(A)** and the wood finger print region **(B)**. Spectra represent average spectra of the clusters according to color code.

Hierarchical cluster analysis (HCA) was further used for the extraction of chemical information of the tissue containing the Eu:HfO_2_ nanocrystals. In a first step, the spectroscopic region for the analysis was chosen to cover the intrinsic emission of the particles (1800–2650 cm^−1^), while the spectral region typical for wood was excluded (Figure 4A). The hyperspectral map was fragmented into 5 clusters, according to differences in intensity and shape of the bands in this spectral region.

The fluorescence signal of the particles was detected across the whole cell wall area. In agreement with the extracted line scans, the cell corners and middle lamellae showed an intense particle luminescence. An even higher intensity of the particles was detected at the interface between lumen and cell wall. Considering only particle clustering (Figure 4A), the lowest intensity was found in the inner part of the S2 and in the lumen close to the interface. In order to investigate whether the particle presence coincides with regions of specific chemical composition in the wood structure, the same clustering algorithm was applied to the spectral range 1100–1700 cm^−1^, the spectral region containing various characteristic vibrations of the polymeric components in the cell wall, mainly cellulose, lignin and hemicelluloses (Figure 4B).

The aromatic ring stretching, with the characteristic band around 1600 cm^−1^, representing the phenolic compounds, has the highest intensity. Therefore, the applied clustering algorithm yielded a fragmentation into chemically differing regions with strong dependence on lignin content. The most prominent band for cellulose at 1095 cm^−1^ confirms this clustering. As expected, cellulose content is lowest in the cluster resembling cell corner and compound middle lamella (dark blue), where the lignin concentration is the highest.

A relatively high signal associated with the particle luminescence was also detected at the interface between the cell wall and the lumen, with no direct counterpart in the clustering corresponding to a wood cell wall component. This suggests that a layer of particles is present at the interface between cell wall and lumen, sticking to the cell wall but not being incorporated into the wood structure.

**Figure 5:**
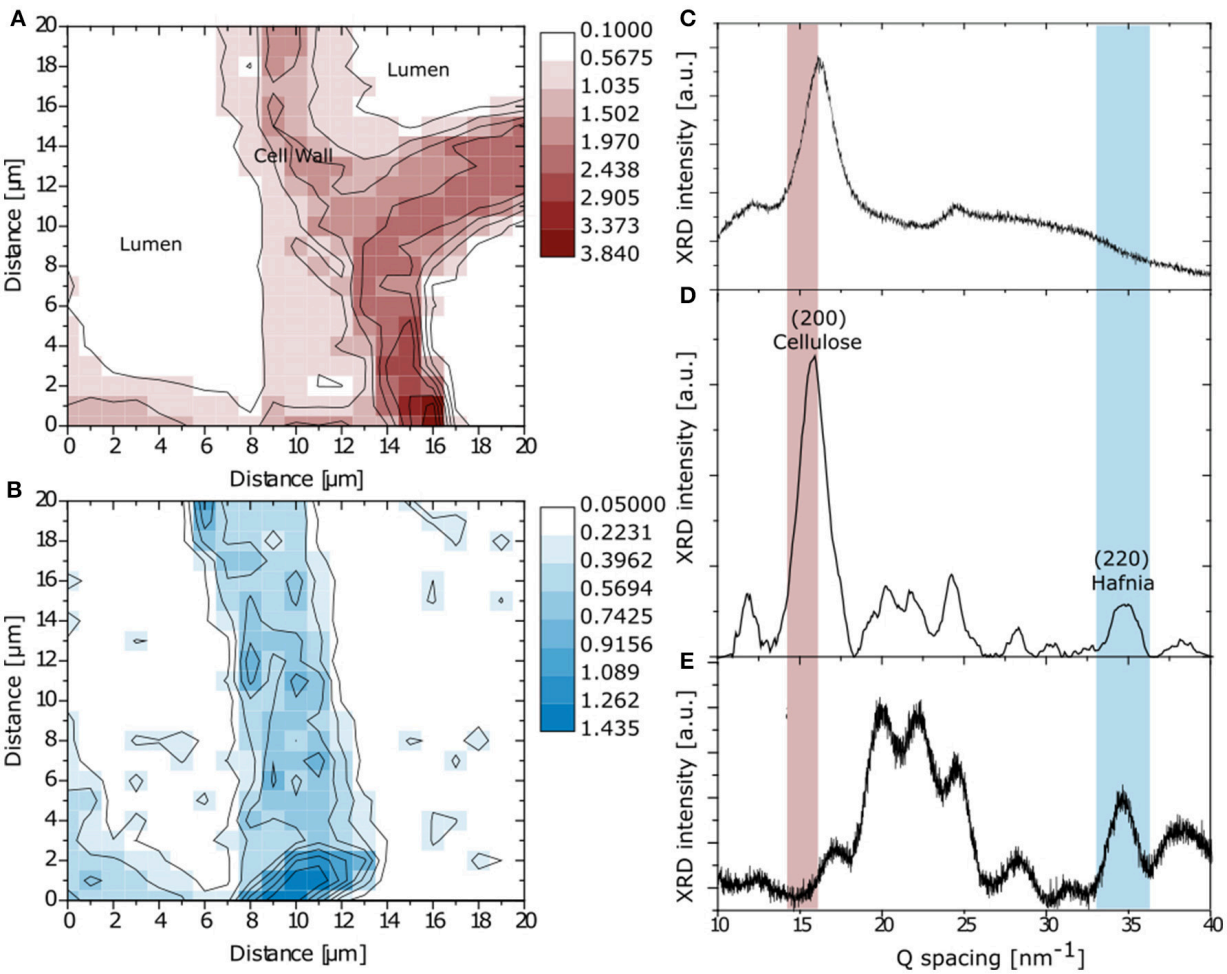
Mapping of integrated intensity in Q-space over main cellulose reflection (200) **(A)** and Hafnia (220) **(B)**. Reference XRD pattern for spruce **(C)** and monoclinic Eu:HfO_2_ **(E)**. Diffraction profile extracted from the mapping of the particle infiltrated wood sample **(D)**.

For an additional confirmation of the particle presence in the cell wall, transmission micro-focused synchrotron WAXS was performed by scanning particle infiltrated wood cross-sections. Figures 5C,E display the diffraction profiles of the pure components, i.e., untreated spruce wood and monoclinic Eu:HfO_2_ particles, respectively, obtained through laboratory powder diffraction which are compared to one profile extracted from a mapping of the particle infiltrated wood sample (Figure 5D), recorded at the synchrotron.

An integration in Q-space over the main cellulose reflection (200) (Figure 5A) and the (220) reflection of monoclinic hafnia (Figure 5B) was conducted for simultaneous recognition of wood structure and hafnia incorporation [JCPDS: 01-078-0050] (Nishiyama et al., 2002). The spatial reconstruction of the obtained maps confirms the successful diffusion of the Hafnia nanoparticle sol into the cell wall structure and is in good agreement with the Raman microscopy analysis. Nevertheless, on the right side of the map, the cell wall resembled from the reflection of crystalline cellulose does not show any presence of hafnia nanoparticles (Figure 5B), clearly discriminating between infiltrated and non-accessed cells. This is in accordance with the SEM investigation, where the presence of matter of high electron density was found in cells adjacent to empty cells (Figure 2).

## Discussion

The distribution patterns of particles observed with the three complementary analytical techniques further raise the fundamental question regarding the relevant diffusion pathways of water based media in the wood structure. Main locations of particle agglomeration were found at the lumen interface and in the middle lamella/cell corner area.

A comparison between the two obtained mappings shows correlation between high particle concentration and the chemical composition of the different anatomical regions in the cell wall. Accumulation of particles in lignin rich areas (compound middle lamella) indicates a stronger affinity of the particles to lignin compared to cellulose or hemicelluloses. Moreover, in accordance with the interaction of particles with the highly lignified middle lamella, it should be noted that the secondary cell wall layer S3 was decorated with particle clusters (Figure 2). The S3 is the terminal layer toward the lumen and is known to possess a higher lignin content compared to the other secondary cell wall layers (Donaldson, 1987). The particle accumulation in lignin rich areas can be explained by chemical interactions of the methoxy groups of the stabilizing ligand of the particles with functional groups on the phenolic units of lignin. This indication of possible specific interactions of the surfactant affecting the particle distribution in the cell wall might be used in future studies to accumulate the nano-tracker system during the diffusion process in areas with a specific chemical functionality. The simultaneous detection of the particles and vibrational signatures of the surrounding tissue in a single Raman measurement can deliver further insight into the given molecular organization of biological materials.

The lumina, particularly of the early wood tracheids, constitute the main water pathway in the spruce wood structure at the microscale (Zimmermann and Brown, 1971). Since the particles are in aqueous solution during the infiltration, they can diffuse into the nano-pores of the water swollen cell wall. Subsequent drying results in a reduction of the free volume in the cell wall and the pore system (Papadopoulos et al., 2003). The free diffusion pathways for the particles are consequently diminished, and particles are physically trapped in the cell wall and hence, they could also be detected, although at lower concentration in the secondary cell wall. Accordingly, the particles could diffuse from the lumen into the cell wall and reach the compound middle lamella through the secondary cell wall layer. However, this seems rather unlikely in view of the lower concentration of particles in the S2 layer without any gradient. Fengel and Wolfsgruber, who infiltrated small cubes of pine wood with aqueous solutions of metal compounds and studied the distribution pattern of the stain in a transmission electron microscope, suggested a second pathway system, running along the interconnected middle lamellae and cell corners (Fengel et al., 1971). In their study, an increased concentration of metal compounds was found in the compound middle lamellae, at the interface between lumen and cell wall and at the bordered pit structures. The concentration in the secondary cell wall, especially the S2 region, was lower. The present results make it reasonable to assume that both pathways, schematically visualized in Figure 1B, are relevant for wood infiltration and transport processes in plant materials and most probably in the living plant.

Resolving transport pathways in plant tissues requires a detailed knowledge on the diffusion patterns and accessibility of different cell wall areas. In this study a novel ultra-small nanoparticle system was utilized as a nano-tracker to study transport pathways and penetration in wood tissue. Macroscopic wood samples have been infiltrated with a stable aqueous suspension of Eu:HfO_2_ nanocrystals. The properties of these nanocrystals, i.e., crystallinity, high electron density, and tailored optical properties allowed for their detection by electron microscopy, micro focus WAXS, and confocal Raman microscopy. All methods confirmed the presence of the nano-trackers throughout the whole macroscopic sample, and their successful incorporation into the cell wall. The simultaneous collection of the vibrational signature of wood and the tailored fluorescence of the nanocrystals in a single Raman measurement enables to combine spatial distribution of the nanoparticles with chemical information of the targeted tissue. From the obtained distribution pattern, it can be concluded, that particulate matter with an average size smaller than 5 nm can be incorporated into the native cell wall structure of wood.

## Conclusion

A penetration throughout the whole thickness of the macroscopic wood structures with nanoparticles of ultra-small size was successfully conducted in this diffusion-based infiltration study. The aqueous suspension of hafnia nanoparticles was effectively impregnated in the hierarchical structure, reaching the hollow regions of the cell lumina as well as the nano-scaled porosity of the cell wall and the interconnecting layer between the single cells.

The simultaneous collection of the vibrational signature of wood and the tailored fluorescence of the nanocrystals in a single Raman measurement enables to combine spatial distribution of the nanoparticles with chemical information of the targeted tissue. Based on this detailed study on the presence of the nanoparticles in distinct anatomical regions, possible pathways for their integration can be suggested. A system of open porosity, percolating through the cell wall structure and into the middle lamella and cell corner region can therefore be assumed.

A better understanding of transport pathways in plant tissues is crucial for industrial processing of lignocellulosic materials, but also fundamental for the targeted functionalization of biological materials for the development of novel bio-based composites (Keplinger et al., 2016; Leitch et al., 2016; Li et al., 2016). With the possibility to tailor the surface chemistry of the particles, an investigation of specific interactions and functionalities in the native assembly of plant tissue, even in subcellular structures of porous and rigid biological materials can be envisaged. The employment of such nano-scale probes as a contrast agent in non-specific and specific targeting experiments for bio-imaging of cellular and subcellular transport pathways could reveal further insight into the ultrastructure of wood as well as of other rigid but porous biological materials.

## Author contributions

JS designed the study with support of AL. JS planned the experiments and JS, AL, TK, JB performed experiments and analyzed data. JS, TK, AL, JB, and IB co-wrote the paper. JS drew the conclusions. All authors discussed results and commented on the manuscript. All authors read and approved the final manuscript.

### Conflict of interest statement

The authors declare that the research was conducted in the absence of any commercial or financial relationships that could be construed as a potential conflict of interest.
